# Disease of Women Unconnected with Pregnancy

**Published:** 1853-09

**Authors:** 


*Disease of Women Unconnected with Pregnancy. Final Cause of Menstruation.* Dr. F. Ramsbotham has published a short paper enunciating views which have given rise to considerable discussion. He admits to the fullest extent the ovular theory of Bischoff and Pouchet; but, in addition, he propounds the hypothesis that the menstrual secretion and the dicidua are convertible phenomena, the one or the other occurring according as impregnation does or does not take place. This identity of the two products, he thinks, is established by the following considerations —

An ovule, he observes, ripe for impregnation, parts from the ovarium and is grasped by the Fallopian fimbriæ. At the same time, nature establishes an action in the uterus for the purpose of preserving it, provided it becomes impregnated. In this case the fluid formed is retained in the uterus, and becomes gradually converted into deciduous membrane. If, on the contrary, for want of impregnation the ovule perishes, then this fluid, being no longer required, is allowed to pass away and becomes the menstrual fluid.

This view, the author thinks, is strengthened by the fact that the menstrual fluid and the decidua seem both to be the product of the same tubular glands; that the decidua, when first formed, is a viscid fluid; and that, in dysmenorrhea, a membrane is not unfrequently formed in the virgin uterus, which has very much the external characters of decidua; that those females who menstruate irregularly or painfully are not so obnoxious to pregnancy as those in whom the function is normally performed; and that in the lower animals, in which there is no menstruation, there is no deciduous membrane.—*Half Yearly Abstract of the Medical Sciences.*

